# Nutritional status and risk of contrast-associated acute kidney injury in elderly patients undergoing percutaneous coronary intervention

**DOI:** 10.1007/s10157-021-02061-4

**Published:** 2021-04-12

**Authors:** Xiaoqi Wei, Hanchuan Chen, Zhebin You, Jie Yang, Haoming He, Chen He, Weiping Zheng, Kaiyang Lin, Feng Jiang

**Affiliations:** 1 Department of Geriatric Medicine, Shengli Clinical Medical College of Fujian Medical University, Fujian Provincial Hospital, Fujian Provincial Institute of Clinical Geriatrics, Fujian Key Laboratory of Geriatrics, Fujian Provincial Center for Geriatrics, Fuzhou, 350001 Fujian China; 2 Department of Cardiology, Shengli Clinical Medical College of Fujian Medical University, Fujian Provincial Hospital, Fujian Provincial Key Laboratory of Cardiovascular Disease, Fuzhou, 350001 Fujian China

**Keywords:** Malnutrition, Controlling Nutritional Status (CONUT) score, Contrast-associated acute kidney injury, Percutaneous coronary intervention, Elderly

## Abstract

**Background:**

This study aimed to investigate the connection between malnutrition evaluated by the Controlling Nutritional Status (CONUT) score and the risk of contrast-associated acute kidney injury (CA-AKI) in elderly patients who underwent percutaneous coronary intervention (PCI).

**Methods:**

A total of 1308 patients aged over 75 years undergoing PCI was included. Based on the CONUT score, patients were assigned to normal (0–1), mild malnutrition (2–4), moderate-severe malnutrition group (≥ 5). The primary outcome was CA-AKI (an absolute increase in ≥ 0.3 mg/dL or ≥ 50% relative serum creatinine increase 48 h after contrast medium exposure).

**Results:**

Overall, the incidence of CA-AKI in normal, mild, moderate-severe malnutrition group was 10.8%, 11.0%, and 27.2%, respectively (*p* < 0.01). Compared with moderate-severe malnutrition group, the normal group and the mild malnutrition group showed significant lower risk of CA-AKI in models adjusting for risk factors for CA-AKI and variables in univariate analysis (odds ratio [OR] = 0.48, 95% confidence interval [CI]: 0.26–0.89, *p* = 0.02; OR = 0.46, 95%CI: 0.26–0.82, *p* = 0.009, respectively). Furthermore, the relationship were consistent across the subgroups classified by risk factors for CA-AKI except anemia. The risk of CA-AKI related with CONUT score was stronger in patients with anemia. (overall interaction *p* by CONUT score = 0.012).

**Conclusion:**

Moderate-severe malnutrition is associated with higher risk of CA-AKI in elderly patients undergoing PCI.

**Supplementary Information:**

The online version contains supplementary material available at 10.1007/s10157-021-02061-4.

## Introduction

With the development of coronary angiography, more and more elderly patients with coronary heart disease receive percutaneous coronary intervention (PCI). However, elderly patients with coronary diseases who underwent percutaneous coronary intervention (PCI), were more likely to develop contrast-associated acute kidney injury (CA-AKI) than general population [1]. CA-AKI is a relatively common complication after intravascular contrast media administration, which significantly prolongs days for hospitalization, increases risk of mortality and morbidity [2]. Since therapeutic strategies for CA-AKI are limited, early screening of this high-risk population and implement preventive measurement are particularly important.

Malnutrition, which is high present in elderly patients [3, 4], is also a predisposing factor for AKI [5, 6]. The Controlling Nutritional Status (CONUT) score, an objective and comprehensive tool for nutrition assessment, is calculated from the serum albumin value, the total cholesterol level, and the total lymphocyte count [7]. The prognostic value of the CONUT score has been proved in patients with coronary artery disease [8, 9]. However, previous studies emphasized on the outcomes of mortality and adverse cardiovascular events. The role of the CONUT score in CA-AKI, one of the adverse outcomes after PCI, has not been investigated in elderly patients undergoing PCI.

It has been demonstrated that each component of the CONUT score is related to kidney injury [5, 10, 11]. Therefore, we hypothesized that the CONUT score was associated with the incidence of CA-AKI in elderly patients undergoing PCI. We aim to evaluate the predictive value of the CONUT score for CA-AKI.

## Methods

### Study population

**Fig. 1:**
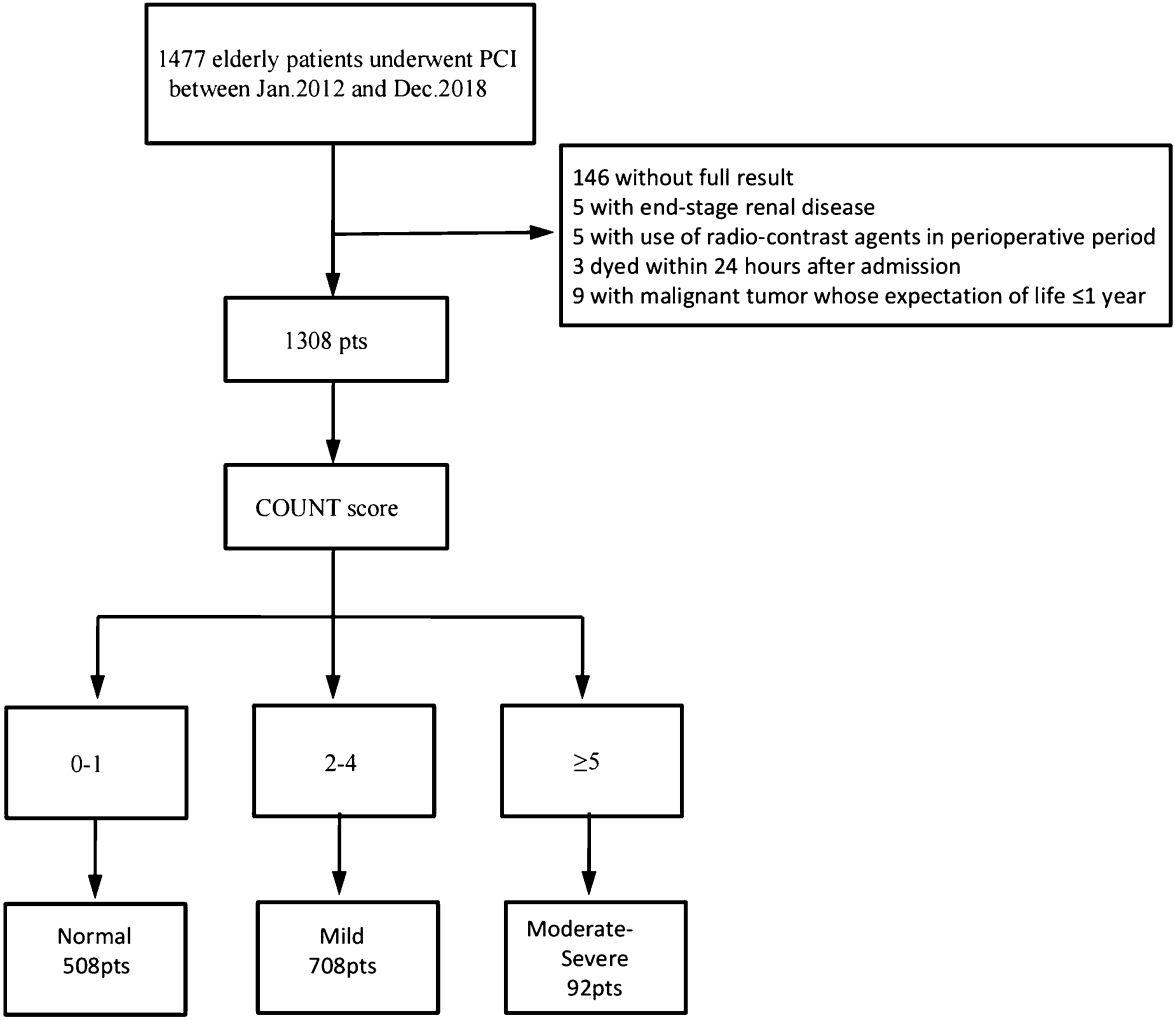
Study population

We conducted a retrospective cohort study at the Fujian Provincial Hospital, Fujian Cardiovascular Institute, between January 2012 and December 2018. A total of 1477 elderly patients aged ≥ 75 years undergoing PCI were enrolled. The exclusion criteria were as follows: (1) died within 24 h after admission (*n* = 3); (2) end-stage renal disease (estimated glomerular filtration rate [eGFR] ≤ 15 mL/min/1.73 m^2^; *n* = 5); (3) history of radio-contrast agents 48 h prior to procedure or 72 h post-procedure (*n* = 3); (4) lack of data on pre-procedural or post-procedural serum creatinine (SCr) levels (*n* = 73); (5) lack of data of serum albumin level, the total cholesterol level, or the total lymphocyte count (*n* = 73); (6) history of malignant tumor with expectation of life less than 1 year (*n* = 9). Consequently, 1308 patients were eligible in the study (Fig. 1).

### Protocol

Data collected included basic characteristics [e.g. age, hypertension, diabetes mellitus (DM)] and procedure related data (e.g. number of diseased vessels). The serum albumin level, total cholesterol level, and the lymphocyte count was measured for each patient at admission. SCr was measured at admission and daily for 3 days after contrast exposure. We also measured white blood cell (WBC) count, hemoglobin (HGB), hematocrit (HCT) and other standard clinical parameters on the morning of the first or second day after admission. The eGFR was calculated using the modified modification of diet in renal disease equation [12]: eGFR = 175 × (SCr/88.4)^−1.554^ × age^−0.203^ × 0.742 (if female) × 1.212 (if black). The use of medication was determined by the cardiologists according to clinical protocols based on guidelines. Left ventricular ejection fraction (LVEF) was evaluated using echo-cardiography during hospitalization. PCI was performed by experienced interventional cardiologists. All patients received nonionic contrast media (either Iopamiron or ultravist, both 370 mg/mL). In addition, all patients received 0.9% normal saline (NS) at a rate of 1 mL/kg/h for 12 h during perioperative period(or 0.5 mL/kg/h for 12 h if patients had overt heart failure) [13]. The protocol met the requirements of the Declaration of Helsinki and was approved by the ethics committee of the Fujian Provincial Hospital, China (ethics approval number: K2012-01-011).

### Nutrition status evaluation

Nutritional status was evaluated based on the CONUT score, which includes values of serum albumin, total cholesterol level, and total lymphocyte count, with a score ranging from 0 to 12 (Table S1) [7]. Higher scores indicates a worse nutritional status. Based on the CONUT score, patients were divided into normal (0–1), mild malnutrition (2–4), moderate-severe (≥ 5) malnutrition groups. The classification of nutritional status has also been applied in other studies [14, 15].

### Definitions and end points

The primary end point was the development of CA-AKI, defined as an absolute increase in ≥ 0.3 mg/dL or ≥ 50% from the baseline SCr levels within 48 h after exposure to contrast media (CM) [16, 17]. Anemia was defined as HCT < 0.39 (for males) or, < 0.36 (for females). The diagnosis of myocardial infarction (MI) is detection of cardiac troponin I (cTnI) above the 99th percentile upper reference limit (URL) and with one of the following: symptoms of ischaemia; new or presumed new significant ST-segment-T wave (ST-T) changes or new left bundle branch block (LBBB); development of pathological Q waves in the ECG; identification of an intracoronary thrombus by angiography [18].

### Statistical analyses

All statistical analyses were performed using R version 4.0.2. The baseline characteristics were compared among three groups divided by CONUT score. Normally distributed continuous variables were expressed as mean ± standard deviation (SD). The Student's *t*-test, Wilcoxon rank sum test was performed to determine the differences between CA-AKI and Non CA-AKI groups. And one way-analysis of variance was performed to determine the differences between groups classified by CONUT score. The categorical variables were represented as percentages and analyzed using chi-square test or Fisher’s exact test.

After testing for proportional odds assumptions, multivariate logistic analysis was used to examine the association of CONUT score 0–1 and CONUT score 2–4 (vs. CONUT score ≥ 5) with CA-AKI in models adjusted as follows: model 1 adjusted for traditional risk factors for CA-AKI (age, anemia, DM, contrast media(CM) volume > 200 ml, eGFR < 60 mL/min/1.73m^2^); and model 2 adjusted for variables in model 1 plus the variables with *p* value < 0.05 in the univariate statistical results including AF, emergency PCI, MI, perioperative hypotension, WBC, blood glucose, hyperuricemia (HUA). Interactions between the primary end point and prespecified subgroups stratified by several CA-AKI risk factors were assessed using a likelihood ratio test for interaction. The *p* values for interaction were calculated in each subgroup. A 2-sided *p* value < 0.05 was considered as statistically significant.

## Result

### Baseline characteristics

A total of 1308 elderly patients were included in this study. Baseline characteristics are listed in Table 1. By CONUT calculations, 508 (38.8%) patients were not malnourished, 708 (54.1%) patients had mild malnutrition, 92 (7.0%) patients had moderate-severe malnutrition, respectively. Patients with moderate-severe malnutrition were more likely to have MI, emergency PCI, anemia, hyperuricemia. They also had lower level of WBC, lymphocyte count, serum albumin, HCT, total cholesterol, but higher level of glucose, higher percentage of eGFR < 60 mL/min/1.73 m^2^ (all *p* < 0.05).

**Table 1:** Baseline characteristics of patients in different groups classified by CONUT score

Variables	Normal (CONUT 0–1)	Mild (CONUT 2–4)	Moderate-severe (CONUT ≥ 5)	*p* Value
	*N* = 508	*N* = 708	*N* = 92	
*Demographics*				
Age, years	78.89 3.34	79.09 3.55	80.66 4.48	< 0.001
Sex, female, *n* (%)	194 (38.19%)	152 (21.47%)	20 (21.74%)	< 0.001
Systolic blood pressure, mmHg	138.24 23.40	134.44 22.05	127.30 20.62	< 0.001
Diastolic blood pressure, mmHg	72.27 12.43	71.55 23.58	67.94 12.71	0.155
Hypertension, *n* (%)	377 (74.2%)	552 (78.0%)	74 (80.4%)	0.211
Diabetes, *n* (%)	175 (34.5%)	260 (36.7%)	37 (40.2%)	0.498
Smoker, *n *(%)	140 (30.2%)	229 (36.2%)	27 (32.9%)	0.114
Atrial fibrillation, *n* (%)	62 (12.2%)	102 (14.4%)	12 (13.0%)	0.536
Emergency PCI, *n *(%)	68 (13.4%)	109 (15.4%)	25 (27.2%)	0.003
Myocardial infarction, *n *(%)	191 (37.6%)	304 (42.9%)	67 (72.8%)	< 0.001
Perioperative hypotension, *n* (%)	45 (8.9%)	76 (10.7%)	18 (19.6%)	0.009
*Laboratory measurements*				
Serum creatinine, μmol/L	84.46 28.86	89.69 30.60	108.09 85.05	< 0.001
WBC, × 10^9^/L	7.74 2.44	7.39 2.66	7.78 3.45	0.049
lymphocyte, × 10^9^/L	2.04 0.58	1.51 0.58	1.02 0.40	< 0.001
HGB, g/L	132.1514.92	129.34 16.13	117.07 20.98	< 0.001
HCT	0.39 0.04	0.38 0.04	0.35 0.06	< 0.001
ALB, g/L	40.73 3.33	39.46 4.04	33.27 4.36	< 0.001
Cholesterol, mg/dL	189.88 39.51	43.70 32.91	121.97 27.93	< 0.001
Glucose, mmol/L	7.12 2.97	7.23 3.08	9.02 4.82	< 0.001
Uric acid, μmol/L	380.68 102.36	377.61 108.65	354.82 121.18	0.103
eGFR, mL/min/1.73 m^2^	80.37 24.70	78.93 25.36	70.17 25.31	0.002
eGFR < 60 mL/min/1.73 m^2^, *n* (%)	100 (19.7%)	156 (22.0%)	31 (33.7%)	0.011
Urine PH	6.27 0.71	6.29 0.74	6.25 0.72	0.885
Anemia, *n *(%)	181 (35.6%)	331 (46.8%)	65 (70.7%)	< 0.001
Hyperuricemia, *n*(%)	203 (40.0%)	243 (34.3%)	26 (28.3%)	0.035
*Medical therapy during hospitalization*				
Statin, *n *(%)	498 (98.0%)	699 (98.7%)	91 (98.9%)	0.637
ACEI/ARB, *n *(%)	405 (79.7%)	586 (82.8%)	71 (77.2%)	0.242
Antiplatelet, *n* (%)	498 (98.0%)	695 (98.2%)	89 (96.7%)	0.598
Metformin, *n* (%)	73 (14.4%)	87 (12.3%)	12 (11.0%)	0.539
*Procedure characteristic*				
Multi-vessel coronary artery disease, *n *(%)	403 (79.3%)	569 (80.4%)	78 (84.8%)	0.479
Number of diseased vessels, *n *(%)	2.32 ± 0.82	2.38 ± 0.82	2.45 ± 0.75	0.254
Number of stents, *n *(%)	1.59 ± 0.75	1.68 ± 0.80	1.65 ± 0.74	0.160
Iso-osmolar contrast media use, *n *(%)	172 (33.9%)	267 (37.7%)	34 (37.0%)	0.381
Volume of contrast media, mL	177.07 52.39	177.70 57.76	179.78 54.41	0.909
Contrast volume > 200 mL, *n *(%)	88 (17.3%)	128 (18.1%)	19 (20.7%)	0.741

*WBC* white blood cell, *HGB* hemoglobin, *HCT* hematocrit, *ALB* albumin, *eGFR* estimated glomerular filtration rate, *PCI* percutaneous coronary intervention, *ACEI* angiotensin-converting enzyme inhibitor, *ARB* angiotensin receptor blocker

And baseline characteristics between CA-AKI group and non CA-AKI group are presented in Table 2. Based on the CONUT score, 25 (15.8%) patients in CA-AKI group and 67 (5.8%) patients in non CA-AKI group had moderate-severe malnutrition, respectively. Patients who developed CA-AKI were more likely to have MI, emergency PCI, DM, AF, HUA, as well as higher level of WBC, blood glucose, lower level of serum albumin and lymphocyte count. More patients in the CA-AKI group were more likely to be treated with contrast volume of ≥ 200 mL (all *p* < 0.05).

**Table 2:** Baseline characteristics between non CA-AKI group and CA-AKI group

	Non CA-AKI	CA-AKI	*p* Value
	*N* = 1150	*N* = 158	
*Demographics*			
Age, years	79.0 53.49	79.70 4.06	0.121
Sex, female, *n* (%)	311 (27.0%)	55 (34.8%)	0.052
Systolic blood pressure, mmHg	135.96 22.21	131.64 25.47	0.078
Diastolic blood pressure, mmHg	71.66 19.93	71.03 13.88	0.723
Hypertension, *n* (%)	877 (76.3%)	126 (79.8%)	0.384
Emergency PCI, *n*(%)	153 (13.3%)	49 (31.0%)	< 0.001
Diabetes, *n* (%)	590 (51.3%)	109 (69.0%)	< 0.001
Atrial fibrillation, *n* (%)	142 (12.4%)	34 (21.5%)	0.002
Smoker, *n* (%)	352 (34.0%)	44 (30.4%)	0.430
MI, *n *(%)	455 (39.6%)	107 (67.7%)	< 0.001
Perioperative hypotension, *n* (%)	96 (8.3%)	43 (27.2%)	< 0.001
*Medical therapy during hospitalization*			
Statin, *n* (%)	1132 (98.4%)	156 (98.7%)	1.000
Antiplatelet agents, *n* (%)	1129 (98.3%)	153 (96.8%)	0.231
ACEI/ARB, *n* (%)	931 (81.0%)	123 (77.9%)	0.413
Metformin, *n* (%)	150 (13.0%)	21 (13.3%)	1.000
*Laboratory measurements*			
Serum creatinine, μmol/L	88.09 29.88	95.27 69.28	0.597
WBC, × 10^9^/L	7.39 2.48	8.72 3.42	< 0.001
Lymphocyte, × 10^9^/L	1.70 0.64	1.54 0.69	0.005
HGB, g/L	129.96 16.06	126.71 19.03	0.099
HCT	0.38 0.05	0.37 0.05	0.073
ALB, g/L	39.78 4.15	37.57 4.21	< 0.001
Cholesterol, mg/dL	159.85 43.50	161.98 38.06	0.187
Glucose, mmol/L	7.17 3.07	8.38 4.01	< 0.001
eGFR, mL/min/1.73 m^2^	78.93 23.75	78.47 34.06	0.247
eGFR < 60 mL/min/1.73 m^2^, *n* (%)	242(21.0%)	45 (28.5%)	0.044
Urine PH	6.29 0.72	6.22 0.80	0.090
CONUT score			< 0.001
0–1, *n *(%)	453 (39.4%)	55 (34.8%)	
2–4, *n *(%)	630 (54.8%)	78 (49.4%)	
≥ 5, *n *(%)	67 (5.8%)	25 (15.8%)	
Anemia, *n* (%)	267 (23.2%)	48 (30.4%)	0.061
Hyperuricemia, *n*(%)	395 (34.4%)	77 (48.7%)	0.001
*Procedure characteristic*			
Contrast volume, mL	176.14 55.91	188.23 51.01	0.006
Contrast volume ≥ 200 mL, *n* (%)	190 (16.5%)	45 (28.5%)	0.003
Iso-osmolar contrast media use, *n* (%)	416 (36.2%)	57 (36.1%)	1.000
Number of stents, *n* (%)	1.65 0.78	1.58 0.73	0.388
Multi-vessel coronary artery disease, *n* (%)	915 (79.57%)	135 (85.44%)	0.102

*MI* myocardial infarction, *PCI* percutaneous coronary intervention, *ACEI* angiotensin-converting enzyme inhibitor, *ARB* angiotensin receptor blocker, *eGFR* estimated glomerular filtration rate, *ALB* albumin, *WBC* white blood cell, *HGB* hemoglobin, *HCT* hematocrit

### Risk factors of CA-AKI

The incidence of CA-AKI was 10.83%, 11.02%, 27.17%, from the group of CONUT 0–1 to the group of CONUT ≥ 5, respectively (*p* < 0.001) (Table 3). After adjusting for traditional risk factors for CA-AKI, such as age, anemia, DM, CM > 200 ml, eGFR < 60 mL/min/1.73 m^2^, multiple logistic regression analysis confirmed that the group of CONUT score 0–1 and the group of CONUT score 2–4 were associated with a lower risk of CA-AKI after PCI, compared with the group of CONUT score ≥ 5 (odds ratio[OR] 0.40, 95% confidence interval [CI] 0.23–0.72, *p* = 0.002; OR 0.40, 95%CI 0.23–0.70, *p* = 0.001) (Table 3). After adjusting for variables in model 1 plus the other variables including AF, emergency PCI, MI, perioperative hypotension, WBC, blood glucose, hyperuricemia(HUA), the group of CONUT score 0–1 and the group of CONUT score 2–4 remained significant lower risk of CA-AKI in elderly patients after PCI, compared with the group of CONUT score ≥ 5 (OR 0.48, 95%CI 0.26–0.89, *p* = 0.02; OR 0.46, 95%CI 0.26–0.82, *p* = 0.009) (Table 3). In other words, CONUT score ≥ 5 was independently associated with a higher risk of CA-AKI, compared with CONUT score 0–1 and CONUT score 2–4.

**Table 3:** Associations between CONUT score and CA-AKI

	Participants, *n*	Events, *n*	Rate,%	Model 1^*^ OR (95%CI)	*p* Value	Model 2^†^ OR (95%CI)	*p* Value
CONUT score 0–1	508	55	10.83	0.40 (0.23–0.72)	0.002	0.48 (0.26–0.89)	0.02
CONUT score 2–4	708	78	11.02	0.40 (0.23–0.70)	0.001	0.46 (0.26–0.82)	0.009
CONUT score ≥ 5	92	25	27.17	Reference	–	Reference	–

^*^Model 1 adjusted for age, anemia, diabetes, contrast media volume > 200 ml, eGFR < 60 ml/(min·1.73 m^2^). ^†^Model 2 adjusted for variables in model 1 plus atrial fibrillation, emergency PCI, MI, WBC, glucose, hyperuricemia, perioperative hypotension. *CI* confidence interval, *OR* odds ratio

**Fig. 2:**
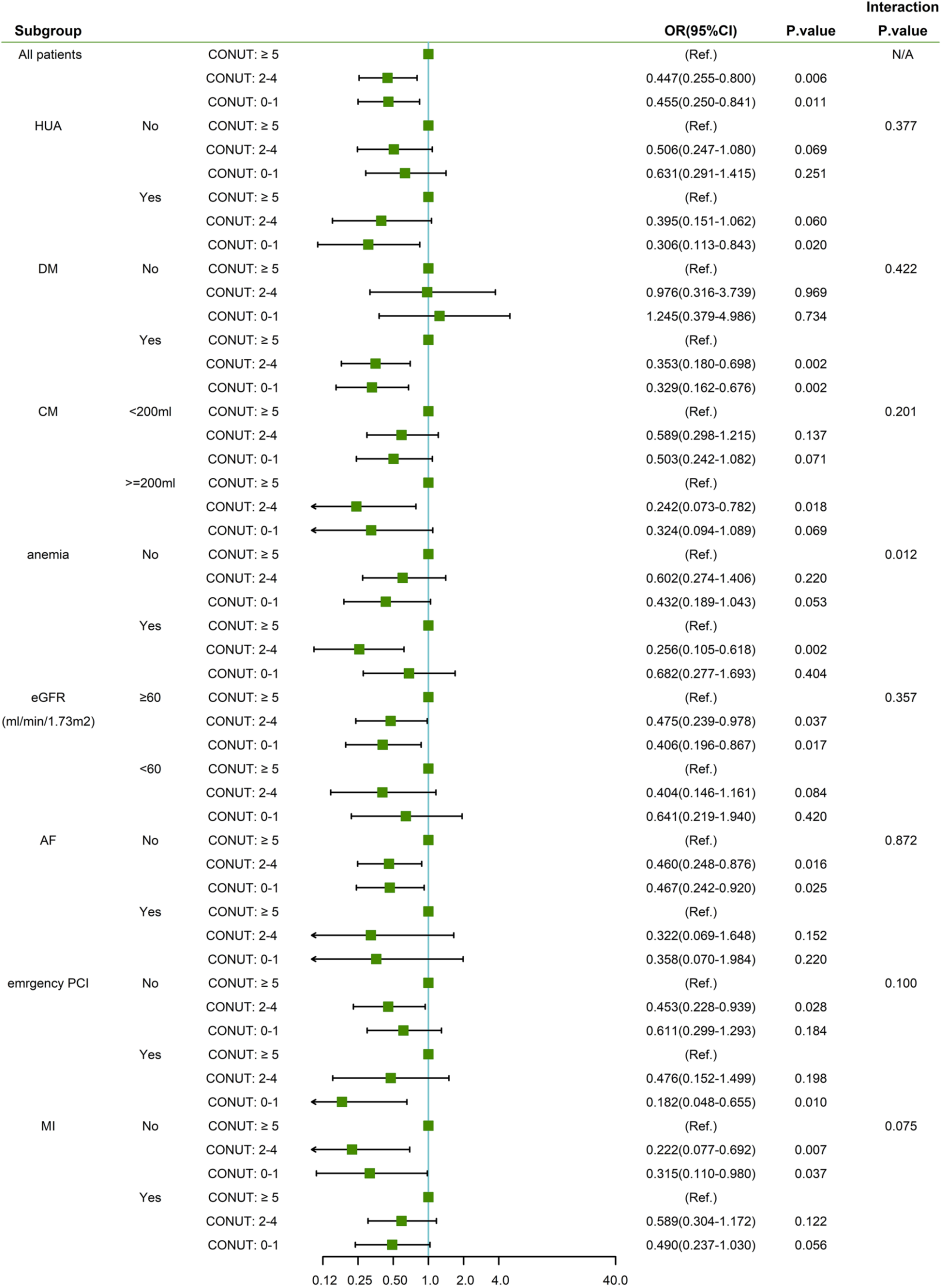
Subgroup analysis of the effect of CONUT score on CA-AKI incidence in the matched cohort

**Fig. 3:**
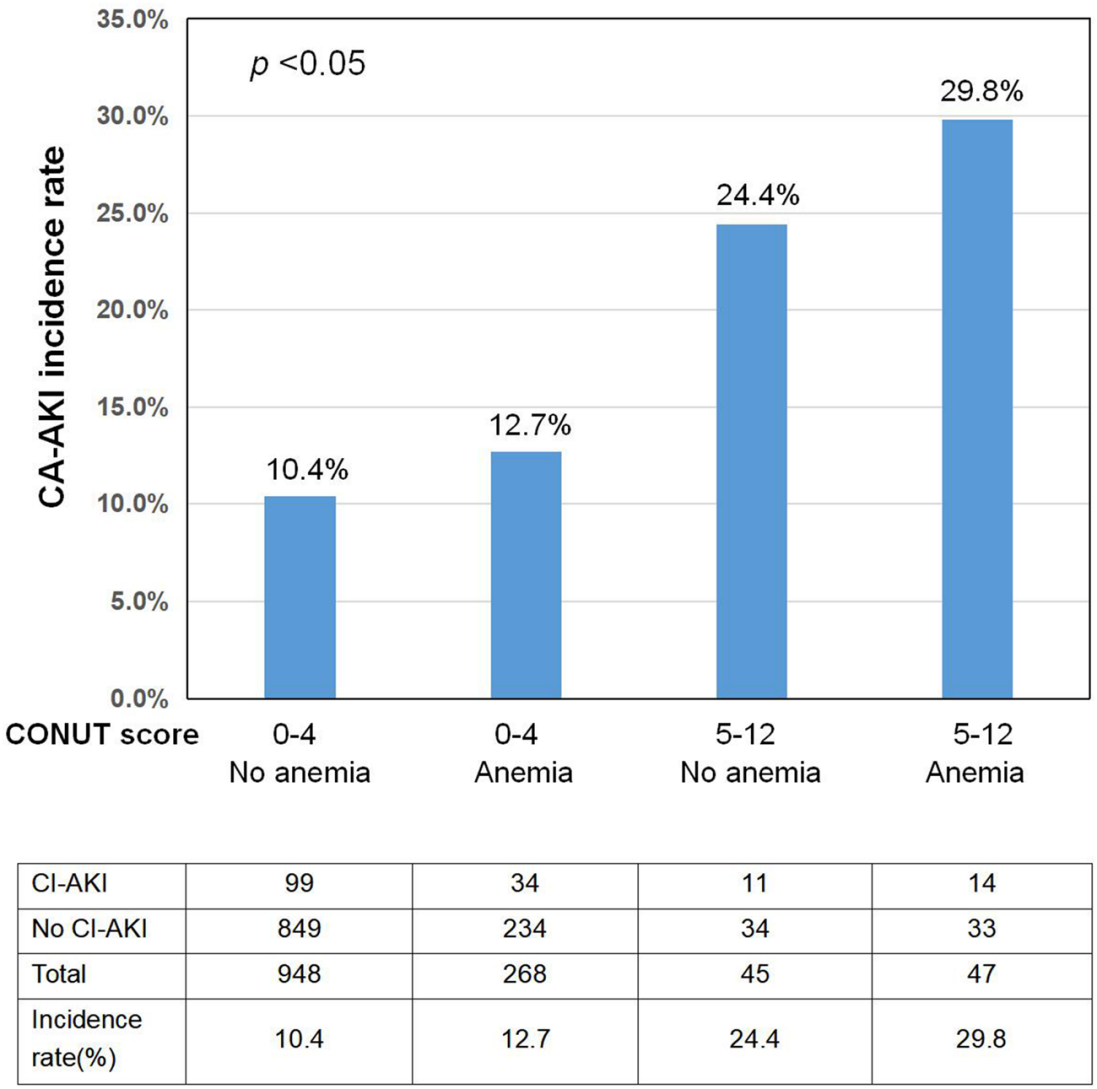
Incidence rate of CA-AKI in patients with various combination of CONUT score and anemia

The effects of the CONUT score on the rate of CA-AKI were consistent across the prespecified subgroups (HUA, DM, CM, eGFR, AF, emergency PCI, MI) (Fig. 2). However, there was an modification by anemia: the risk of CA-AKI related with CONUT score was stronger in patients with anemia than in those without anemia (overall interaction *p* by CONUT score = 0.012) (Figs. 2, 3).

## Discussion

To our knowledge, this is the first study to demonstrate the relationship between the objective nutrition scoring tool “CONUT” and the incidence of CA-AKI in elderly patients undergoing PCI. Our results show that the CONUT score ≥ 5, recognized as moderate or severe malnutrition, is associated with an increased risk of CA-AKI in elderly patients undergoing PCI. Moreover, the association is stronger in elderly patients with anemia.

In our study, the incidence of CA-AKI in elderly patients undergoing PCI was up to 12.1%, which was almost consistent with the data available in a meta-analysis [19]. Age over 75 years was widely recognized as an independent risk factor of CA-AKI in patients after PCI [1]. It’s well-known that comorbidities are high present in the admitted elderly patients [20], such as the age-related decrease in kidney function [21] and anemia [22], which were identified as risk factors of CA-AKI [1, 23]. Due to multiple chronic diseases, the elderly patients often take multiple oral drugs, which may worsen kidney function. Moreover, as poor vascular and heart condition, adequate hydration is difficult to achieve and thus nephrotoxic contrast cannot be discharged as soon as possible. Therefore, the elderly is more vulnerable. It’s important to effectively further identify elderly patients at higher risk for CA-AKI and implement precise prevention.

In addition to the above factors, malnutrition is high present in elderly patients [3], which is also associated with the development of kidney injury [24]. However, the best tool to identify patients at high risk of malnutrition is still in dispute. Single nutrition indicators are often affected by many factors [25]. Subjective comprehensive nutritional scoring systems like Subjective Global Assessment (SGA), are sophisticated, which need the assistance of specialized nutritionists. But there are some objective comprehensive nutritional scoring systems such as the CONUT score, Prognostic Nutritional Index (PNI), which only require simple blood biomarkers and make it convenient to apply in clinic. The CONUT score is calculated from the serum albumin value, the total cholesterol level, and the total lymphocyte count [7], which only included one more indicator than PNI. The CONUT score has been used for assessing the prognosis of heart failure [15], acute ischemic stroke [26], and a variety of malignant tumors [27, 28]. Recently, the prognostic value of the CONUT score has been validated and shown better than PNI in patients with coronary artery disease. In an observational, retrospective study of 3118 cohort, patients undergoing PCI were divided into four groups by their CONUT score (0–1 vs. 2–4 vs. 5–8 vs. 9–12). It revealed that patients with higher CONUT scores had higher rates of major adverse cardiac events (hazard ratio [HR]: 1.14; 95% CI: 1.07–1.22, *p* < 0.05)[8]. Basta et al. evaluated the CONUT and PNI score in 945 patients with ST-elevation myocardial infarction undergoing PCI and found that patients with severe CONUT but not patients with severe PNI index had the highest rate for all-cause death, with a log-rank of *p* < 0.001[9]. Roubín et al. made a similar conclusion that the CONUT score has a higher sensitivity than the PNI for all-cause death and major adverse cardiovascular events (MACEs) in patients with acute coronary syndrome [29]. Therefore, it was appropriate to apply the CONUT score in our study. However, these studies emphasized on the outcomes such as mortality and adverse cardiovascular events in patients after PCI. The role of the CONUT score in CA-AKI, which is one of the adverse outcomes of PCI, has not been investigated in elderly patients.

Our study first revealed that elderly patients with moderate-severe malnutrition (CONUT score ≥ 5) had higher risk of CA-AKI. Even after adjusting for multiple risk factors, moderate-severe malnutrition is also significantly associated with the development of CA-AKI. Our subgroup analysis also confirms the relationship between the CONUT score and the risk of CA-AKI, although there is an modification by anemia. This could probably be explained by the added risk of anemia to CA-AKI. It was recognized that anemia on admission is associated with an increased risk of CA-AKI [1]. The possible explanation may be the anemia-induced aggravation of renal ischemia and hypoxic injury to the outer medulla in the kidney [30]. Combined with malnutrition, the capacity of scavenging oxygen free radical is reduced, which may aggravate cell toxicity and further impair the kidney function [31].

The mechanism of the connection between the CONUT score and CA-AKI has not been fully investigated. However, each component of CONUT score has been demonstrated the relationship with kidney injury. First, serum albumin levels, which are twice the weight of other indicators in CONUT score, can predict the risk of CA-AKI. A meta-analysis including about 68,000 subjects confirmed that lower serum albumin is an independent predictor both of AKI and death after AKI in patients undergoing cardiac surgery or acute coronary interventions [6]. Furthermore, a retrospective study found that in patients treated with PCI, the serum albumin level was significantly lower in the CA-AKI group (3.52 + 0.40 vs. 3.94 + 0.39 mg/dL, *p* < 0 0.001) and it was an independent predictor of CA-AKI[5]. Second, although hypercholesterolemia is a well-established risk factor for cardiovascular disease in the general population [32], the correlation between low serum cholesterol and adverse outcomes has been reported for patients with renal failure. Obialo et al. evaluated a 3-year retrospective study of patients with acute kidney injury and demonstrated that survival was higher among patients with cholesterol > 150 mg/dL than those whose levels were < or = 150 mg/dL (*p* < 0.001) [10]. Another study also showed that lower total cholesterol (TC) levels over time were significantly associated with worse survival (HR 1.66, 95% CI 1.11–2.47) in patients with chronic kidney diseases who underwent peritoneal dialysis [33]. Finally, lymphocyte count may represent a marker of the inflammation response [34], which is a significant and crucial factor in the pathogenesis of CA-AKI [35]. A low relative lymphocyte count was shown to be independently associated with worse prognosis in patients with CAD [36]. Additionally, several studies have shown that higher ratio of neutrophil/lymphocyte is related to a greater risk of CA-AKI in patients who underwent PCI [11, 37], suggesting that a low lymphocyte count may contribute to the development of CA-AKI. Thus, the CONUT score, reflecting not only the status of malnutrition but also the degree of inflammation, may be more appropriate for early detection of elderly patients with high risk of CA-AKI.

### Limitation

First, this study was a single-center cohort with a relatively small sample size. A potential patient selection bias may be existed. Second, the CONUT score was evaluated only at the time of admission, and we did not assess the effect of the change of score during the observation period. Thirdly, elderly patients comprised major proportion of participants in our study. Therefore, our results may not be applied to younger patients. Furthermore, based on this study, it is unclear whether the stratified risk classes need an nutritional intervention.

## Conclusion

We find that moderate-severe malnutrition is strongly associated with high risk of CA-AKI in elderly patients who underwent PCI. Moreover, the risk of CA-AKI related with CONUT score was stronger in patients with anemia. Further studies are required to determine whether nutrition support improves clinical outcomes in this population.

## Supplementary Information

Below is the link to the electronic supplementary material.

Supplementary file1 (DOC 27 KB)

## References

[1] Mehran R, Aymong ED, Nikolsky E (2004). A simple risk score for prediction of contrast-induced nephropathy after percutaneous coronary intervention: development and initial validation. J Am Coll Cardiol.

[2] McCullough PA, Choi JP, Feghali GA (2016). Contrast-induced acute kidney injury. J Am Coll Cardiol.

[3] Ljungqvist O, van Gossum A, Sanz ML, de Man F (2010). The European fight against malnutrition. Clin Nutr.

[4] Kaiser MJ, Bauer JM, Rämsch C (2010). Frequency of malnutrition in older adults: a multinational perspective using the mini nutritional assessment. J Am Geriatr Soc.

[5] Murat SN, Kurtul A, Yarlioglues M (2015). Impact of serum albumin levels on contrast-induced acute kidney injury in patients with acute coronary syndromes treated with percutaneous coronary intervention. Angiology.

[6] Wiedermann CJ, Wiedermann W, Joannidis M (2017). Causal relationship between hypoalbuminemia and acute kidney injury. World J Nephrol.

[7] Ignacio de Ulíbarri J, González-Madroño A, de Villar NG (2005). CONUT: a tool for controlling nutritional status. First validation in a hospital population. Nutr Hosp..

[8] Chen SC, Yang YL, Wu CH (2020). Association between preoperative nutritional status and clinical outcomes of patients with coronary artery disease undergoing percutaneous coronary intervention. Nutrients.

[9] Basta G, Chatzianagnostou K, Paradossi U (2016). The prognostic impact of objective nutritional indices in elderly patients with ST-elevation myocardial infarction undergoing primary coronary intervention. Int J Cardiol.

[10] Obialo CI, Okonofua EC, Nzerue MC, Tayade AS, Riley LJ (1999). Role of hypoalbuminemia and hypocholesterolemia as copredictors of mortality in acute renal failure. Kidney Int.

[11] Yuan Y, Qiu H, Hu X (2017). Predictive value of inflammatory factors on contrast-induced acute kidney injury in patients who underwent an emergency percutaneous coronary intervention. Clin Cardiol.

[12] Levey AS, Coresh J, Greene T (2006). Using standardized serum creatinine values in the modification of diet in renal disease study equation for estimating glomerular filtration rate. Ann Intern Med.

[13] Isaka Y, Hayashi H, Aonuma K (2020). Guideline on the use of iodinated contrast media in patients with kidney disease 2018. Clin Exp Nephrol.

[14] Naito H, Nezu T, Hosomi N (2018). Controlling nutritional status score for predicting 3-mo functional outcome in acute ischemic stroke. Nutrition.

[15] Agra Bermejo RM, González Ferreiro R, Varela Román A (2017). Nutritional status is related to heart failure severity and hospital readmissions in acute heart failure. Int J Cardiol.

[16] Miura T, Miyashita Y, Motoki H (2017). Efficacy and safety of percutaneous coronary intervention for elderly patients in the second-generation drug-eluting stent era: the SHINANO Registry. Angiology.

[17] Davenport MS, Perazella MA, Yee J (2020). Use of intravenous iodinated contrast media in patients with kidney disease: consensus statements from the American College of Radiology and the National Kidney Foundation. Radiology.

[18] Thygesen K, Alpert JS, Jaffe AS (2012). Third universal definition of myocardial infarction. Eur Heart J.

[19] Song W, Zhang T, Pu J, Shen L, He B (2014). Incidence and risk of developing contrast-induced acute kidney injury following intravascular contrast administration in elderly patients. Clin Interv Aging.

[20] Fabbian F, De Giorgi A, Maietti E (2017). A modified Elixhauser score for predicting in-hospital mortality in internal medicine admissions. Eur J Intern Med.

[21] Glassock RJ, Rule AD (2016). Aging and the kidneys: anatomy, physiology and consequences for defining chronic kidney disease. Nephron.

[22] Eisenstaedt R, Penninx BW, Woodman RC (2006). Anemia in the elderly: current understanding and emerging concepts. Blood Rev.

[23] Chen H, He C, You Z (2021). Association between urine pH and risk of contrast-associated acute kidney injury among patients after emergency percutaneous coronary intervention: a V-shape relationship. Clin Exp Nephrol..

[24] Li C, Xu L, Guan C (2020). Malnutrition screening and acute kidney injury in hospitalised patients: a retrospective study over a 5-year period from China. Br J Nutr.

[25] Berbel MN, Pinto MP, Ponce D, Balbi AL (2011). Nutritional aspects in acute kidney injury. Rev Assoc Med Bras (1992)..

[26] Cai ZM, Wu YZ, Chen HM (2020). Being at risk of malnutrition predicts poor outcomes at 3 months in acute ischemic stroke patients. Eur J Clin Nutr.

[27] Ahiko Y, Shida D, Horie T (2019). Controlling nutritional status (CONUT) score as a preoperative risk assessment index for older patients with colorectal cancer. BMC Cancer.

[28] Hirahara N, Tajima Y, Fujii Y (2019). Controlling Nutritional Status (CONUT) as a prognostic immunonutritional biomarker for gastric cancer after curative gastrectomy: a propensity score-matched analysis. Surg Endosc.

[29] Raposeiras Roubín S, Abu Assi E, Cespón Fernandez M (2020). Prevalence and prognostic significance of malnutrition in patients with acute coronary syndrome. J Am Coll Cardiol.

[30] Kim SJ, Salem MR, Joseph NJ, Madayag MA, Cavallino RP, Crystal GJ (1990). Contrast media adversely affect oxyhemoglobin dissociation. Anesth Analg.

[31] Taverna M, Marie AL, Mira JP, Guidet B (2013). Specific antioxidant properties of human serum albumin. Ann Intensive Care.

[32] Mach F, Baigent C, Catapano AL (2020). ESC/EAS Guidelines for the management of dyslipidaemias: lipid modification to reduce cardiovascular risk. Eur Heart J.

[33] Park CH, Kang EW, Park JT (2017). Association of serum lipid levels over time with survival in incident peritoneal dialysis patients. J Clin Lipidol.

[34] Bergquist J, Tarkowski A, Ewing A, Ekman R (1998). Catecholaminergic suppression of immunocompetent cells. Immunol Today.

[35] Seeliger E, Sendeski M, Rihal CS, Persson PB (2012). Contrast-induced kidney injury: mechanisms, risk factors, and prevention. Eur Heart J.

[36] Bian C, Wu Y, Shi Y (2010). Predictive value of the relative lymphocyte count in coronary heart disease. Heart Vessels.

[37] Zorlu C, Koseoglu C (2020). Comparison of the relationship between inflammatory markers and contrast-induced nephropathy in patients with acute coronary syndrome after coronary angiography. Angiology.

