# Supplementary material for: Nutritional status and risk of contrast-associated acute kidney injury in elderly patients undergoing percutaneous coronary intervention
Source: Clin Exp Nephrol. 2021 Apr 12;25(9):953–62. doi: 10.1007/s10157-021-02061-4 (PMC8357678; doi:10.1007/s10157-021-02061-4)
Supplement: Supplementary file 1 — Supplementary file1 (DOC 27 KB) [file 10157_2021_2061_MOESM1_ESM.doc]

**Title**

Nutritional status and risk of contrast-associated acute kidney injury in elderly patients undergoing percutaneous coronary intervention

**Journal name**

Clinical and Experimental Nephrology

**Authors and affiliations**

Xiaoqi Wei^1,#^, Hanchuan Chen^2,#^ , Zhebin You^1^, Jie Yang^1^, Haoming He^2^, Chen He^2^, Weiping Zheng^1^, Kaiyang Lin^2,*^, Feng Jiang^1,*^

^1^Department of Geriatric Medicine, Shengli Clinical Medical College of Fujian Medical University, Fujian Provincial Hospital, Fujian Provincial Institute of Clinical Geriatrics, Fujian Key Laboratory of Geriatrics, Fujian Provincial Center for Geriatrics, Fuzhou, Fujian 350001, China;

^2^Department of Cardiology, Shengli Clinical Medical College of Fujian Medical University, Fujian Provincial Hospital, Fujian Provincial Key Laboratory of Cardiovascular Disease, Fuzhou, Fujian 350001, China;

# These authors contributed equally to this work.

***Corresponding Authors**

Kai yang Lin: lky7411@sina.com[;](mailto:sycdxy66@163.com;) Feng Jiang: jiangfengconut@126.com

Table S1: Evaluation of CONUT score

| Parameter | Score |  |  |  |
| --- | --- | --- | --- | --- |
| Serum albumin, g/dL | ≥3.5 | 3.0-3.4 | 2.5-2.9 | <2.5 |
| Albumin score | 0 | 2 | 4 | 6 |
| Total cholesterol, mg/dL | ≥180 | 140-179 | 100-139 | <100 |
| Cholesterol score | 0 | 1 | 2 | 3 |
| Total lymphocytes, count/mL | ≥1600 | 1200-1599 | 800-1199 | <800 |
| Lymphocyte score | 0 | 1 | 2 | 3 |

CONUT, Controlling Nutritional Status.
